# Effect of Combined Manual Therapy and Therapeutic Exercise Protocols on the Postural Stability of Patients with Non-Specific Chronic Neck Pain. A Secondary Analysis of Randomized Controlled Trial

**DOI:** 10.3390/jcm11010084

**Published:** 2021-12-24

**Authors:** Carlos Bernal-Utrera, Ernesto Anarte-Lazo, Juan Jose Gonzalez-Gerez, Manuel Saavedra-Hernandez, Elena De-La-Barrera-Aranda, Maria Angeles Serrera-Figallo, Maribel Gonzalez-Martin, Cleofas Rodriguez-Blanco

**Affiliations:** 1 Physiotherapy Department, Faculty of Nursing, Physiotherapy and Podiatry, University of Seville, 41009 Seville, Spain; cbutrera@us.es (C.B.-U.); cleofas@us.es (C.R.-B.); 2 Fisiosur I + D Research Institute, Garrucha, 04630 Almeria, Spain; clinicafisiosur@gmail.com (J.J.G.-G.); fisioelenacordoba@gmail.com (E.D.-L.-B.-A.); 3 Doctoral Program in Health Sciences, University of Seville, 41009 Seville, Spain; anartelazo.ernesto@gmail.com; 4 Department Nursing, Physiotherapy and Medicine, Faculty of Almeria, 04120 Almeria, Spain; 5 Morphological and Socio-Health Sciences Department, University of Cordoba, 14071 Cordoba, Spain; 6 Stomatology Department, Faculty of Dentistry, University of Seville, 41009 Seville, Spain; migm@us.es

**Keywords:** neck pain, chronic pain, exercise therapy, musculoskeletal manipulations, postural stability

## Abstract

Postural stability is a little-studied factor in non-specific chronic neck pain; the causes that can alter it are unknown. The relationship with chronic pain could be a determining factor for its deficit. The aim of this study was to investigate the relationship between sustained pain and a postural stability deficit. A randomized and blinded clinical trial (double-blind; placebo control; 12 weeks follow-up) was conducted with a total of 69 subjects divided into three groups, two experimental (manual therapy and specific exercise) and a control treatment, and carried out over a treatment period of three weeks with a follow-up after 12 weeks. Their postural stability was assessed through the overall balance index (OBI). The postural stability of subjects with non-specific chronic neck pain improved in the experimental treatments. There were no statistically significant differences between the experimental groups. This trial found that manual therapy and therapeutic exercise significantly improved OBI compared to the control group. Trial registration: Brazilian Clinical Trial Registry, RBR-2vj7sw.

## 1. Introduction

Non-specific chronic neck pain (NCNP) is a severe condition in many patients whose functional capabilities are reduced and is a significant issue for healthcare systems globally [1,2]. The underlying mechanisms of maintenance, recurrence, and progression of NCNP are not clear. Still, it could be associated with a deficit and alteration of the neck muscles’ proprioception that plays a decisive role in cervical joint position, head motor control, and postural stability (PS) [3,4,5]. Indeed, patients with NCNP usually have alterations in cervical proprioception and postural stability, which is defined as the ability to maintain an upright position [3,6] and relies on the power of the central nervous system (CNS) to correctly identify and selectively focus on the multisensory afferent input [7].

A disturbance in the cervical proprioceptive system may trigger symptoms such as dizziness or vertigo because of connections between this system and the visual and vestibular systems [8,9]. That is, abnormal cervical afferent inputs may cause dizziness, unsteadiness, visual disturbances, and/or postural instability, as neck structures generate proprioceptive signals interpreted by the central nervous system to tune the spatial orientation of the head and, in the last instance, posture control [3,10,11].

This phenomenon has been assessed previously, and it was found that patients with NCNP and post-traumatic neck pain suffer greater drunkenness and fainting sensations and a lack of proprioception than patients with benign paroxysmal vertigo [12]. Furthermore, other studies associate the loss of PS with dysfunction of the upper cervical spine and its musculature, changes in cervical mechanoreceptors and the state of weakness of the musculature [13,14,15]. However, this may not necessarily be associated with traumatic events since these types of alterations have been identified in subjects with NCNP without exposure to trauma [15]. Furthermore, altered muscle coordination patterns are present in patients with whiplash and insidious neck pain [16]. Similarly, with a disturbance in muscle contractile efficiency, postural control is negatively affected [17,18], which also happens in the case of neck muscle fatigue [19].

However, this is not the only theory developed to explain how this multifactorial system can be altered. Some studies indicate that there is a proprioceptive disturbance due to sustained exposure to pain that affects PS through the CNS; these changes may be due to changes in the cortical representation and the interaction between mechanical, central, and peripheral mechanisms [20,21,22], as altered postural stability has also been observed in patients with low back pain [23]. Nonetheless, much remains unknown about why dizziness is present in the chronic neck population and, in general, about the etiology of chronic neck pain [24]. In addition, the implementation of physical therapy has shown important changes on the parasympathetic nervous system, which generates a decrease in the perception of pain, in sustained pain processes [25,26].

The effectiveness of manual therapy and therapeutic exercise in patients with NCNP has been proven in numerous studies to improve pain, disability, and dizziness [27,28,29,30]. However, how these therapies influence postural stability has not been verified, and there is very little evidence in this field [31]. Since the relationship between neck pain and postural stability is still unknown, new findings are needed.

Our study aims to analyze and compare the effects of two experimental treatments that help to reduce pain through different mechanisms of action, and to evaluate the evolution of cervical pain and its relationship with changes produced on postural stability. We hypothesize that both treatments will decrease neck pain intensity and that these changes will be related to improvements in postural stability.

## 2. Materials and Methods

### 2.1. Trial Design

The randomized clinical trial was designed as a controlled, parallel, double-blinded, three-arm clinical treatment trial.

### 2.2. Sample Selection

Individuals with NCNP were recruited through a text message broadcast on Seville (Spain) social networks and were selected based on the eligibility criteria listed below. The study took place at the facilities of the physiotherapy department of the University of Seville.

### 2.3. Inclusion Criteria

Patients between 18–50 years old with current neck pain, with pain having been experienced over the last three months.

### 2.4. Exclusion Criteria

Exclusion criteria included: irradiated neck pain or pain associated with vertigo; diagnosed psychological disorders; radiological findings such as vertebral fractures, tumors, osteoporosis, or others; a history of neck surgery; red flags such as night pain, severe muscle spasm, loss of involuntary weight, symptom mismatch as unexplained symptoms outside of the clinical context; and other physiotherapeutic or pharmacological treatments continued in the previous twelve weeks.

### 2.5. Interventions

#### 2.5.1. Group 1: Manual Therapy

**Figure 1:**
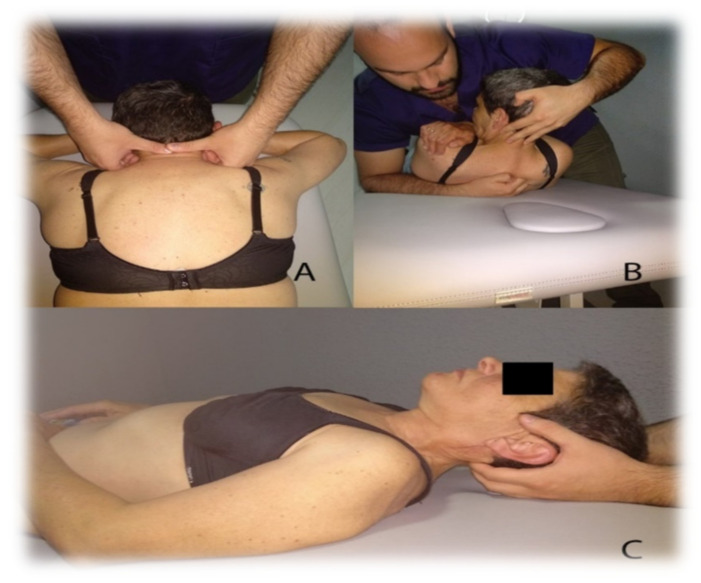
Manual Therapy Protocol. (**A**) Cervical Articular Mobilization, (**B**) High Thoracic Manipulation on T4, (**C**) Suboccipital Muscle Inhibition.

The manual therapy protocol was composed of three techniques based on scientific evidence for neck pain treatment [32,33,34]. This protocol was applied in the three treatment sessions, one per week. The techniques can be seen in Figure 1.

1. High Thoracic Manipulation on T4. Patients are positioned supine with their arms crossed in a “V” shape over the chest. The therapist makes contact with the fist at the level of the spinous process of T4 and blocks the patient’s elbows with his chest; following this, he introduces flexion of the cervical spine until a slight tension is felt in the tissues the point of contact. Downward and cranial manipulation is applied. If cavitation is not achieved on the first attempt, the therapist repositions the patient and performs a second manipulation. A maximum of two attempts will be allowed in each patient [32].
2. Cervical Articular Mobilization (2 Hz, 2 min × 3 series). The patient is placed on the stretcher prone, placing both hands under his forehead. The therapist makes contact with his two thumbs on the spinous process of the patient’s C2 vertebra and performs grade III posteroanterior impulses at a speed of 2 Hz and for two minutes. There are three mobilization intervals with a minute of rest between each one of them [33].
3. Suboccipital Muscle Inhibition (3 min). With the patient lying supine, the therapist places both hands under the subject’s head by contacting their fingers on the lower edge of the occipital bone, exerts constant and painless pressure in the anterior and cranial direction for three minutes [34,35].

#### 2.5.2. Group 2: Therapeutic Exercise

**Figure 2:**
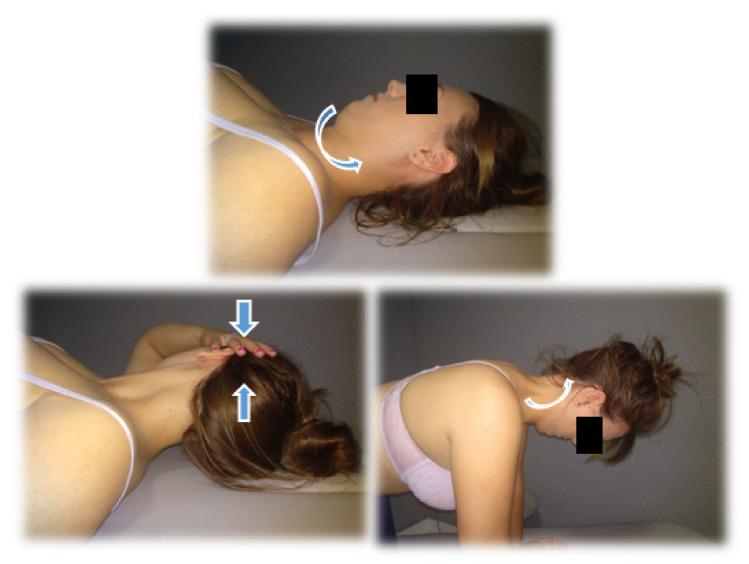
Examples of therapeutic exercises.

Therapeutic Exercise protocol: this protocol is based on a progression in load composed of different phases. At first, activation and recruitment of deep cervical flexors [27]. Secondly, isometric exercise deep and superficial flexors co-contraction [27]. Finally, excentric recruitment of flexors and extensors [27,36,37]. As far as we know, this protocol has not been studied, but activation of this musculature has been observed during tasks similar to our protocol [11,36,37]. This protocol was taught to patients in the first session, which was performed once a day during treatment (three weeks). The physiotherapist reinforced it in each of the three individual sessions. Exercises can be seen in Figure 2.

WEEK 1: Exercises 1 and 2.

1. Cranio-cervical flexion (CCF) in supine position with a towel in the posterior area of the neck. (three sets, 10 repetitions, 10 s of contraction each repetition, and 10 s of rest).
2. CCF sitting. (three sets, 10 repetitions, 10 s of contraction each repetition with 10 s of rest).

WEEK 2: Exercises 1, 2, 3, and 4.

3. Co-contraction of deep and superficial neck flexors in supine decubitus. (10 repetitions, 10 s of contraction with 10 s of rest).
4. Co-contraction flexors, rotators, and inclines. Patients will perform cranial nerve flexion while the physiotherapist asks them to tilt, rotate and look towards the same side while he opposes a resistance with his hand. (10 repetitions, 10 s of contraction with 10 s of rest).

WEEK 3: Exercises 1, 2, 3, 4, 5, and 6.

5. Eccentric for extensors. The patient seated should perform cervical extension; then, they must complete a cranio-cervical flexion and finish doing a cervical flexion (10 repetitions).
6. Eccentric for flexors. The patient will be in a quadrupedal and neutral neck position, should perform neck flexion. They must complete a cranio-cervical flexion and maintain that posture extending the neck and then finally losing the cranio-cervical flexion (10 repetitions).

#### 2.5.3. Group 3: Sham Treatment

Control protocol: Patients were placed in the supine position, while the physiotherapist, sited at the head of the table, sets the palms of his hands under the subject’s head, his fingers contacting the space between the occipital condyle and the spinal process of the second cervical vertebra, but with no pressure at this region, simulating the technique of suboccipital inhibition [35]. After that, a sham laser placebo treatment was applied. This procedure was done with a laser pointer used on the suboccipital musculature for 10 s, with the patient in a prone position and without exerting any pressure. Patients were not informed about the fact that the pointer had been turned off. Patients assigned to the control group received treatment one (manual therapy) or two (therapeutic exercise) after completing the study to avoid ethical bias, so we applied the best treatment to the control group after the study ended.

### 2.6. Outcomes Measures

Outcomes were measured in the pre-evaluation, at week 2 (immediate short-term), week 4 (short-term), and week 12 (medium-term). These evaluations were carried out by a blinded and trained evaluator.

Visual Analog Scale (VAS) for pain. The subjects participating in the study indicated the intensity of their pain using a VAS of 100 mm length, and had to signal on a horizontal line of 100 mm where they would place their pain, where zero mm indicated no pain, and 100 mm indicated the worst pain imaginable [38]. Only differences higher than 15 mm were deemed to have clinical significance [39].

**Figure 3:**
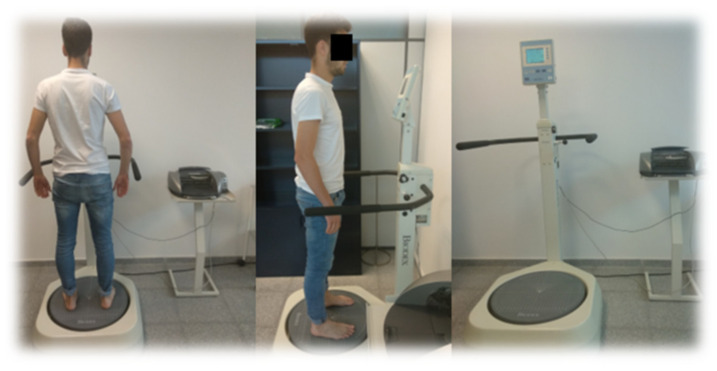
Biodex Balance System ™ SD.

Overall Balance Index (OBI). We obtained this measurement through a dynamic stabilometric platform (Balance System™ SD, Biodex, New York, NY, USA). The general stability test was applied in difficulty four (4), with one (1) being the highest and eight (8) the slightest difficulty. The platform is free in the anterior-posterior and medial-lateral axes; it allows obtaining the OBI through the deviations concerning a zero point established before the test, with the platform stable. Two 20-s tests were performed, with one minute between each test, with the second test score that was chosen for the statistical analysis. The index is calculated through the anteroposterior and medial-lateral relationship + standard deviation [40,41]. Subjects with their eyes open and both feet resting on the platform in line with the shoulders, and with an external rotation between 20°–30° on the midline (where they are comfortable), should try to maintain the most stable position possible in the anteroposterior and medial-lateral axis, an example of the patient’s position can be seen in Figure 3. The patient’s ability to keep the platform stable will determine the OBI. To reach a clinical significance, differences must be higher than 9.8% [42]. The Overall Balance Index showed a good-to-excellent and acceptable reliability measured by the ICC in different studies 0.69 [40] and 0.77 [43].

### 2.7. Sample Size Calculation

The sample size was calculated using the Granmo calculator v.7.12, based on: the analysis of the variance of means, estimation of an alpha risk of 5% (0.05), a beta risk of 10% (0.10), and in a unilateral contrast, a typical deviation of 12% (0.12). The minimum difference to detect was 13.5% (0.135), which was based on the minimum clinically important differences in the VAS [39]. Finally, we included 69 patients who were divided into three groups, each group consisting of at least 20 subjects, overcoming this value to assume the possible loss of follow-up. The rate of follow-up losses was 8%, for which 20 subjects were required in each group, assuming that there were three groups.

### 2.8. Randomization

Subjects were divided into three groups (MT, TE, or C) by a simple randomization process carried out with free software called randomization.com (Available from: http://www.randomized.org/ (accessed on 15 January 2019)). Only the principal investigator and auditor knew the randomization sequence, which was guarded throughout the study and kept hidden from the study participants (patients and evaluators).

### 2.9. Blinding

Evaluators and participants in the study were blinded during the entire process. The evaluators were unaware of the study objectives and the allocation of patients in the study groups. The patients did not know which group they belonged to. The principal investigator and auditor obtained the randomization sequence. No participant in the study had access to the randomization sequence, hidden and saved, to guarantee correct randomization with security.

### 2.10. Statistical Analysis

Within a large-scale clinical trial, with multiple dependent study variables, a specific analysis of the variables related to stability and pain, and their correlations, was performed.

The statistical analysis was carried out using the IBM-SPSS Statistics 24 software. The normality test applied to all the variables was the Kolmogorov–Smirnov test. For the contrast of intergroup hypotheses, the student’s *t*-test for paired variables was involved in the case of parametric distributions and Kruskal–Wallis H for non-parametric distributions. One-factor ANOVA was used for the intergroup hypothesis contrast in parametric distributions and Kruskal–Wallis H for nonparametric distributions. Post hoc analysis was obtained through Bonferroni’s contrast for parametric distributions and Mann–Whitney’s U for nonparametric distributions Spearman’s Rho was used to analyse associations between pain (clinical improvement) and postural stability. The confidence level used was 95% (0.05), and the power of the study was 90% (0.1).

## 3. Results

**Figure 4:**
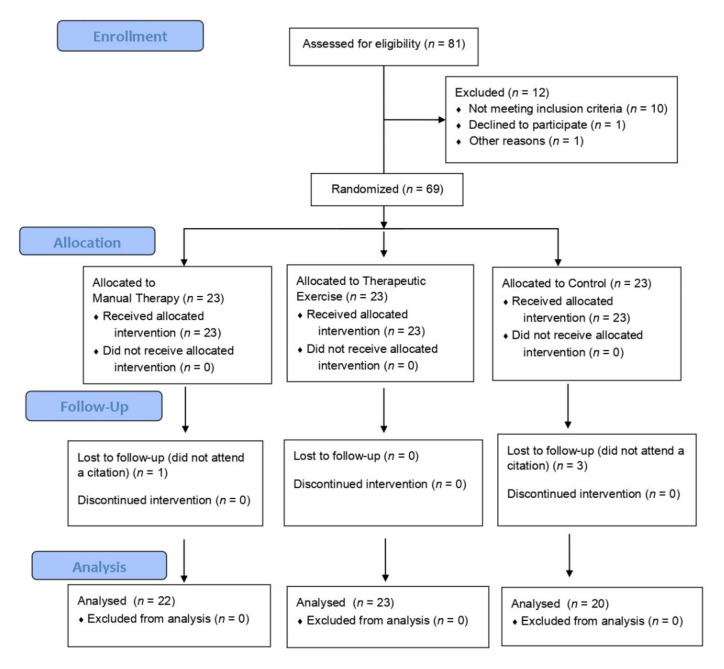
CONSORT Flows Diagram.

Sixty-Nine of the 81 subjects interviewed started the study, of which 65 completed our trial, with a completion rate of 94%. There were four follow-up losses; three of them focused on the control group and one on the manual therapy group. A CONSORT flows diagram is presented in Figure 4.

Participants’ baseline characteristics can be seen in Table 1. The equality of the groups is statistically validated.

The intergroup analysis showed significant improvements for both variables (VAS and OBI) in all evaluations performed in both experimental treatments (MT and TE). The control group did not show differences in VAS and OBI in any assessments. The mean values and their statistical significance are shown in Table 2 and Table 3.

Concerning VAS, the intergroup analysis showed that experimental treatments did not acquire immediate statistically significant results compared to the control group in the short-term. Nonetheless, in the second (week 4) and third evaluations (week 12), statistically significant effects were observed compared to the control group but not between experimental treatments. Improvements were 30.35 [20.98–39.72] in the therapeutic exercise group and 26.14 [1.29–36.98] in a manual therapy group, both in the short-term; in the medium-term, improvements were 23.74 [10.34–37.13] and 23.73 [12.01–35.45], respectively. All of them were higher than 15 and, thus, clinically relevant [35]. There were no significant differences in VAS between the experimental groups in any of the evaluations. 

Regarding the OBI, in the first week of treatment, only the manual therapy group obtained statistically significant differences, obtaining a mean difference with respect to a control of 1.41 [0.32 to 2.50]. However, the intergroup analysis reflected statistically substantial improvements; the intergroup comparison did not get statistically significant results for the therapeutic exercise group when compared to the control group in the first week of treatment, so progress in the TE group was not sufficient to corroborate the hypothesis in the post hoc (Bonferroni). In the short-term (week 4), both experimental treatments achieved statistically significant differences compared to the control group. The OBI mean difference was 2.30 [0.98–3.63] for manual therapy and 0.88 [−0.44–2.19] for therapeutic exercise, exceeding 9.8% of the baseline value, which is the reference value to establish clinical differences [40]. In addition, differences between the experimental groups were not significant (*p* = 0.064), but the mean differences 1.43 [0.13–2.73] exceeded 9.8%, so they could be considered clinically relevant. In the medium-term (week 12), differences were maintained again, obtaining statistical significance in the two experimental groups and placing their difference of means at 1.53 [0.22–2.83] for manual therapy and 1.08 [−0.21–2.38] for therapeutic exercise regarding the control group, maintaining clinical relevance in the medium-term. No significant differences were obtained between the experimental groups, placing their mean difference at 0.44 [−0.84–1.72]. All expressed values are described for a confidence interval of 95%. The values of statistical significance, mean differences and confidence intervals are shown in Table 4. For VAS-OBI correlation, no statistically significant results were obtained; there was no correlation between these two variables.

## 4. Discussion

This randomized and blinded clinical trial examined changes in the short and medium-term in postural stability and pain perceived by non-specific chronic neck pain subjects.

Our results indicate a clear improvement in VAS and OBI in both experimental groups concerning the short- and medium-term. The control group did not obtain improvements in any of the evaluations. In the immediate short-term, there were not sufficiently large differences in terms of pain between the experimental and control groups, which could be explained by the immediate neurophysiological effects of placebo, which has been observed [43,44]. Therefore, we believe that clinical improvements are a result of the experimental treatments. Techniques and exercises that make up our experimental protocols have shown similar neck pain results [27,28,29]. However, changes in postural stability had not been studied using our methods. Nevertheless, our results coincide with other previously published studies that evaluate cervicogenic dizziness, through other methods, on the medium and long-term efficacy of physical therapy interventions based on manual therapy and exercise concerning postural stability and cervicogenic dizziness [45,46,47].

Our central hypothesis was based on an alteration of the postural stability produced by a disorder in the reception of proprioceptive signals by the proprioceptive, vestibular, visual, and central nervous systems. Other authors have already theorized and opined in this regard about this unknown complex [3,4,5,6,20,21,48].

However, the correlation analysis between VAS and OBI clearly shows no correlation between these two variables, which leads us to think that pain and other factors should be considered when improving postural stability.

Our trial carried out two experimental treatments in two subgroups of patients with NCNP, one through manual therapy and neurophysiological effects [49,50,51,52], and the other through therapeutic exercise, involving reorganization in motor patterns and neuromuscular adaptations [22,53]. As we hypothesized, both treatments showed improvements in postural stability, even with two different action mechanisms. At this point, considering our results that showed improvements both in pain and postural stability but the absence of correlation between improvements in pain (VAS) and postural stability (OBI), we position our results on the influence of neurophysiological effects in the CNS and the reorganization in motor patterns and neuromuscular adaptations, not being able to highlight any of them.

On the other hand, some authors have begun to point out the psycho-behavioral aspects that could significantly influence cervicogenic dizziness and the alteration of postural stability, such as anxiety, depression, fear of movement, or pain catastrophizing [4,5,48]. We must bear in mind that these variables are present in a large number of patients suffering from neck pain (with NCNP, post-traumatic neck pain, and sub-acute neck pain) [54,55]. Psychosocial variables such as social environment, catastrophism, and anxiety could influence the perception of these symptoms, as pain does not explain them. However, research in this field is minimal, so firm conclusions cannot be made, and more research is needed.

The main limitations of our study are that the sample size is not too large and that we only measured a variable representative of postural stability, for this variable an ICC analysis has not been performed. Future studies should assess the pain–postural stability relationship through other technical methods to refute or confirm our findings. In addition, in research about this field, we would find it interesting to include additional features, such as psychosocial variables (anxiety, pain catastrophizing, kinesiophobia). Future studies could also check whether the observed stability improvement only occurs in patients with NCNP or other populations with acute/subacute neck pain or whiplash-associated disorders. 

Our findings could have a significant clinical implication, and be useful for clinicians since we provide active and passive treatment protocols which have proven efficacy and could be chosen or combined depending on the patient’s characteristics.

## 5. Conclusions

Both experimental protocols (manual therapy and therapeutic exercise) produce significant improvements (*p* < 0.05) in the visual analogic scale and overall balance index concerning the control group. The correlation between pain and postural stability was not demonstrated.

## Figures and Tables

**Table 1:** Participants’ baseline characteristics according to the study group.

VARIABLES	GROUP			Z
	MT	TE	C	*p*
Age (i)	42.95 ± 2.89	36.78 ± 2.89	36.90 ± 2.89	0.312 ^b^
Gender (ii) (male, female; %)	23 (5/22); 77 (17/22)	22 (5/23); 78 (18/23)	29 (6/21); 71 (15/21)	0.315 ^c^
Body Mass Index (i) (Kg/m)	24.67 ± 1.13	23.8 ± 0.72	24.75 ± 0.75	0.379 ^b^
VAS (i) (mm)	41.95 ± 4.03	48.17 ± 3.48	49.80 ± 3.53	0.237 ^b^
OBI (i)	6.34 ± 0.77	4.95 ± 0.59	5.47 ± 0.64	0.498 ^b^

C: Control Group; MT: Manual Therapy Group; TE: therapeutic exercise group; Z: Shapiro–Wilk normality test; *p*: statistical significance; ^a^: ANOVA; ^b^: H Kruskal–Wallis; ^c^: Chi Cuadrado; (i) dates expressed as means ± standard deviation; (ii) dates expressed as percentages (partial/total); * Indicate statistically significant differences between groups (*p* < 0.05).

**Table 2:** Intergroup analysis for VAS.

	VAS Pre (i)	VAS Week 1 (i)	*p*	VAS Week 4 (i)	*p*	VAS Week 12 (i)	*p*
MT	41.95 ± 4.03	32.77 ± 3.14	0.007 * ^b^	15.82 ± 3.26	0.001 * _b_	18.23 ± 4.33	0.002 * ^b^
TE	48.17 ± 3.48	35.83 ± 3.88	0.008 * ^a^	17.83± 3.42	0.001 * ^b^	24.43 ± 4.70	0.002 * ^b^
C	49.80 ± 3.53	49.25 ± 3.53	0.342 ^a^	50.55 ± 3.54	0.518 ^a^	48.75 ± 3.51	0.337 ^a^

C: control group; MT: manual therapy group; TE: therapeutic exercise group; *p*: statistical significance from tests: ^a^: *t* student for paired samples; ^b^: Z Wilcoxon; (i) dates expressed as means ± standard deviation; * Indicate statistically significant differences between groups (*p* < 0.05).

**Table 3:** Intergroup analysis for OBI.

	OBI Pre (i)	OBI Week 1 (i)	*p*	OBI Week 4 (i)	*p*	OBI Week 12 (i)	*p*
MT	6.34 ±0.77	4.87 ± 0.64	0.001 * ^b^	4.01 ± 0.47	0.001 * ^b^	4.77 ± 0.65	0.003 * b
TE	4.95 ±0.59	3.91 ± 0.41	0.017 * ^b^	4.06 ±0.73	0.013 * ^b^	3.83 ± 0.47	0.010 * b
C	5.47 ±0.64	5.42 ± 0.62	0.808 ^b^	5.47 ± 0.66	0.745 ^b^	5.43 ± 0.62	0.579 b

C: control group; MT: manual therapy group; TE: therapeutic exercise group; *p*: statistical significance from tests: ^b^: Z Wilcoxon; (i) dates expressed as means ± standard deviation; * Indicate statistically significant differences between groups (*p* < 0.05).

**Table 4:** Intergroup Analysis.

VARIABLE	*p*	Post hoc	*p*	*p*	Post hoc	*p*	*p*	Post hoc	*p*
	Week 1			Week 4			Week 12		M and CI
VAS	0.067 ^a^	MT–C	0.0329 ^c^	0.001 ^a^	MT–C	0.001 * ^c^	0.003 ^a^	MT–C	0.008 * ^c^
			7.61			26.71			22.77
			[−3.92 to 19.14]			[12.30 to 41.11]			[4.79 to 40.76]
		TE–C	0.070 ^c^		TE–C	0.001 * ^c^		TE–C	0.007 * ^c^
			10.77			30.92			22.79
			[−0.64 to 22.19]			[16.67 to 45.17]			[5.00 to 40.58]
		MT–TE	1.000 ^c^		MT–TE	1.000 ^c^		MT–TE	1.000 ^c^
			3.17			−4.21			−0.01
			[−14.44 to 8.11]			[−18.29 to 9.87]			[−17.59 to 17.56]
OBI	0.003 * ^b^	MT–C	0.001 * ^d^	0.001 * ^b^	MT–C	0.001 * ^d^	0.013 * ^b^	MT–C	0.006 * ^d^
			1.41			2.30			1.53
			[0.32 to 2.50]			[0.98 to 3.63]			[0.22 to 2.83]
		TE–C	0.116 ^d^		TE–C	0.013 * ^d^		TE–C	0.028 * ^d^
			0.99			0.88			1.08
			[−0.09 to 2.06]			[−0.44 to 2.19]			[−0.21 to 2.38]
		MT–TE	0.261 ^d^		MT–TE	0.064 ^d^		MT–TE	0.420 ^d^
			0.42			1.43			0.44
			[−0.64 to 1.49]			[0.13 to 2.73]			[−0.84 to 1.72]

C: Control Group; MT: Manual Therapy Group; TE: Therapeutic Exercise Group; *p*: Significación Estadística; M: Mean Diference; CI: Confidence Interval; ^a^: ANOVA; ^b^: H Kruskal-Wallis, ^c^: Bonferroni, ^d^: U Mann-Whitney; * Indicate statistically significant differences between groups (*p* < 0.05).

## Data Availability

The data presented in this study are available on request from the corresponding author. The data are not publicly available due to privacy.
